# The role of metabolites under the influence of genes and lifestyles in bone density changes

**DOI:** 10.3389/fnut.2022.934951

**Published:** 2022-09-02

**Authors:** Xuewei Lv, Yanfeng Jiang, Dantong Yang, Chengkai Zhu, Huangbo Yuan, Ziyu Yuan, Chen Suo, Xingdong Chen, Kelin Xu

**Affiliations:** ^1^State Key Laboratory of Genetic Engineering, School of Life Sciences, Human Phenome Institute, Fudan University, Shanghai, China; ^2^Fudan University Taizhou Institute of Health Sciences, Taizhou, China; ^3^Shanghai Municipal Center for Disease Control and Prevention, Shanghai, China; ^4^Ministry of Education Key Laboratory of Public Health Safety, Department of Epidemiology, School of Public Health, Fudan University, Shanghai, China; ^5^Ministry of Education Key Laboratory of Public Health Safety, Department of Biostatistics, School of Public Health, Fudan University, Shanghai, China

**Keywords:** bone mineral density, lifestyle, metabolites, osteopenia, osteoporosis, polygenic risk scores

## Abstract

**Purpose:**

Osteoporosis is a complex bone disease influenced by numerous factors. Previous studies have found that some metabolites are related to bone mineral density (BMD). However, the associations between metabolites and BMD under the influence of genes and lifestyle have not been fully investigated.

**Methods:**

We analyzed the effect of metabolites on BMD under the synergistic effect of genes and lifestyle, using the data of 797 participants aged 55–65 years from the Taizhou Imaging Study. The cumulative sum method was used to calculate the polygenic risk score of SNPs, and the healthful plant-based diet index was used to summarize food intake. The effect of metabolites on BMD changes under the influence of genes and lifestyle was analyzed through interaction analysis and mediation analysis.

**Results:**

Nineteen metabolites were found significantly different in the osteoporosis, osteopenia, and normal BMD groups. We found two high-density lipoprotein (HDL) subfractions were positively associated with osteopenia, and six very-low-density lipoprotein subfractions were negatively associated with osteopenia or osteoporosis, after adjusting for lifestyles and genetic factors. Tea drinking habits, alcohol consumption, smoking, and polygenic risk score changed BMD by affecting metabolites.

**Conclusion:**

With the increased level of HDL subfractions, the risk of bone loss in the population will increase; the risk of bone loss decreases with the increased level of very-low-density lipoprotein subfractions. Genetic factors and lifestyles can modify the effects of metabolites on BMD. Our results show evidence for the precise prevention of osteoporosis.

## Introduction

Osteoporosis is a common polygenic systemic degenerative bone disease characterized by decreased bone mineral density (BMD). More than 200 million people globally are suffering from osteoporosis (1), while one in three women and one in five men over 50 years of age experience osteoporotic fractures, which usually result in a dependent living situation and premature death (2, 3). In China, the prevalence of osteoporosis was 34.65% in people aged over 50 years in 2015 (4). Osteoporosis, a chronic disease caused by numerous factors, urgently needs to attract people’s attention and explore its underlying pathophysiological mechanisms.

Metabolites are referred to as “the link between genotype and phenotype,” which are the downstream output of biological processes, carrying imprints of genomic, epigenomic, and environmental effects. Researchers have shown that there is a correlation between BMD and various lipids and amino acids (5–8), among which many studies suggest that BMD is quantitatively associated with blood lipoprotein levels (9–11). A population-based study in Italy showed that high-density lipoprotein (HDL) is significantly higher in osteoporotic women compared to non-osteoporotic controls (12). Another study reported that reduced HDL levels are associated with the development of an inflammatory microenvironment that affects the differentiation and function of osteoblasts, which in turn may contribute to bone loss (9). Besides, very-low-density lipoprotein (VLDL) has been found associated with bone mass (10), and it has also been suggested to regulate the link between bone loss and vascular calcification (10). One study demonstrated an inverse correlation between serum total cholesterol, low-density lipoprotein cholesterol levels, and BMD (13). However, how metabolites regulate BMD under the synergistic influence of genes and lifestyle remains unclear.

It has been shown that 60–80% of the variation in BMD is inherited from parents and the remainder is derived from the environment (14). *LRP5*, along with *PPARγ* and *ESR1* genes, is reported to regulate both HDL and BMD (3, 15). *LRP5* is a lipoprotein receptor-related protein gene, which is related to HDL and BMD (3, 16). A study has shown that the *LRP5* gene may affect BMD by regulating lipoprotein levels (17). It is reported that *PPARγ* induces the expression of ABCA1, which is involved in the first step of the reverse cholesterol reverse transport pathway and the control of plasma HDL levels (18), and several studies have examined *PPARγ* as a candidate gene for BMD in humans (19, 20). As an estrogen receptor gene, *ESR1* has an impact on the expression or enhanced sensitivity of estrogen action (21). *ESR1* polymorphism was found to be significantly associated with serum concentrations of multiple metabolites including total cholesterol (TC), HDL, and triglyceride (TG) (22–24).

Existing studies have confirmed that osteoporosis is affected by age, sex, waist-to-hip ratio (WHR), lifestyle, etc. (3, 25–28). Central obesity has been proved to be a risk factor for osteoporosis, and WHR is negatively correlated with BMD (27). Several studies have shown that smoking can increase the risk of osteoporosis in many ways (25, 26, 29). In addition, smoking is known to increase total cholesterol, triglycerides, and low-density lipoprotein, while acting to decrease HDL (30). It is reported that a small amount of alcohol consumption may increase bone mass and BMD, but a large amount of alcohol consumption can damage bones at various levels (31). Moreover, alcohol may increase HDL cholesterol (HDL-C) levels and decrease the total LDL cholesterol (LDL-C) (32). Previous studies have also shown that tea consumption could reduce the risk of osteoporosis (33–35). In addition, drinking tea is related to the slower decline of HDL-C concentration with age (36). Moreover, under the plant-based diet, there is a risk of decreased BMD and an increased probability of fractures (37, 38). A plant-based diet reduces calcium and vitamin D intake and has been proved to increase N-telopeptide, which is a biomarker of bone resorption. Therefore, plant-based diets may increase bone resorption (39).

Taizhou Imaging Study is a community-based prospective cohort nested in the Taizhou Longitudinal Study (40), which leverages metabolites, genes, demographics, and lifestyles into the BMD study. In this study, we aim to explore the association between metabolites and BMD, considering the influence of genes and lifestyle habits. We performed association, interaction, and mediation analyses, investigated the association between metabolites and BMD in the Chinese population, and explored the interaction and mediation effects of metabolites played in the relationship between genes, lifestyles, and BMD.

## Materials and methods

### Subject recruitment and study design

**FIGURE 1:**
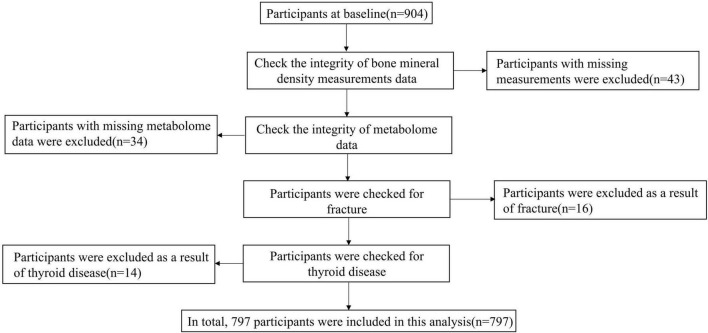
Flowchart of the study design.

All participants in this study were from the Taizhou Imaging Study (41). The Taizhou Imaging Study selected all people aged 55–65 years at enrollment from three natural villages with high compliance and response rates from the Taizhou Longitudinal Study. The inclusion and exclusion criteria of the TIS have been detailed in our previous publication (40). Finally, 904 individuals were included in the baseline study. All participants who completed the baseline survey had completed epidemiological questionnaires (including demographics, lifestyle, disease history, and diet frequency) and physical examination. For this study, 904 individuals who had BMD measurements by DXA scans at baseline were included; among them, 43 were excluded for BMD measurements incomplete. Thirty-four participants were excluded for the missing metabolome data. A total of 16 participants were excluded as a result of a history of fracture. Fourteen participants were excluded as a result of thyroid disease. The final sample size included in the analysis of this study was 797 (Figure 1). The TIS was approved by the Ethics Committee of the School of Life Sciences, Fudan University, and Fudan University Taizhou Institute of Health Sciences (institutional review board approval number: 496 and B017, respectively). Written informed consent was obtained from each participant before data and biospecimen collection.

### Bone mineral density assessment

A dual-energy X-ray absorptiometry (Lunar DPX NT-400157; GE Healthcare, Madison, WI, United States) densitometer was utilized for measuring BMD for the lumbar spine (L1–L4), total hip, and the femoral neck on the left side. In the lumbar spine, BMD was measured in four lumbar vertebrae (L1–L4) and the central portion of a lateral scout view of the first to the fourth vertebra. The BMD (g/cm^2^) of the total hip was an evaluation of BMD in the whole hip bone. The minimum T-score of three measuring positions was used as a threshold to define the three groups based on BMD. The 797 participants were divided into a normal BMD group (*n* = 312) with a T-score of –1 or more, an osteopenia group (*n* = 353) with a T-score between –1 and –2.5, and an osteoporosis group (*n* = 132) with a T-score of –2.5 or less at the lumbar spine, total hip, or femoral neck (42). The measurements were taken in strict accordance with the manufacturer’s instructions for use of the equipment and were evaluated by the same experienced physician using the same machine (43).

### Metabolome information collection and quality control

Untargeted baseline serum metabolomic profiling was performed using the nuclear magnetic resonance (NMR) platform. For each sample, three one-dimensional spectra were acquired, including the NOESYPR1D sequence, Carr-Purcell-Meiboom-Gill sequence, and diffusion-edited spectrum. In addition, a series of two-dimensional spectra were collected from pooled samples for spectral assignment. The high-throughput NMR metabolomic platform could generate spectra from which about 350 metabolic features and ratio parameters will be simultaneously quantified as absolute concentrations of each feature or calculated as ratios, including lipids, lipoproteins, amino acids, and other metabolites, but in this study, only absolute quantitative indicators were used for analysis. All the analyzed serums were not subject to a second freeze–thaw cycle before metabolomic analysis (40). Before the analysis, the metabolites with a missing percentage greater than 20% were removed. For small-molecule metabolites, missing values were replaced by half of the lowest detection limit. Data of under-volume undetected and severe hypoxemia were deleted. After standardizing to Z-scores by an inverse-normal transformation, the metabolite levels of a total of 136 metabolites were normally distributed (44, 45).

### Genomic information collection and polygenic risk score

Genomic DNA was extracted and purified from the whole blood of consenting participants at baseline and genotyped on an Axiom Precision Medicine Research Array (PMRA) (46). Genome-wide imputation grid markers of about 800,000 custom single nucleotide polymorphisms (SNPs) were selected, and clinically relevant variants, pharmacogenomic, functional, and other disease-related variants were included in the PMRA (40). We searched for SNPs related to lipoprotein and BMD in three databases, including GWAS Catalog (47), MetaCore (48), and PhenoScanner (49). The filtered SNPs were matched to the Taizhou genome data, and a total of nine SNP loci were screened out. These nine SNP sites came from two genes, which are *LRP5* (lipoprotein receptor-related protein) and *ESR1* (estrogen receptor 1). The SNPs rs3736228 and rs599083 are on *LRP5*, while the SNPs rs1999805, rs2504069, rs2504063, rs2982552, rs7772579, rs851975, and rs9322341 are on *ESR1*. The cumulative sum method was used to calculate the polygenic risk scores (PRS): The response value of the SNP on each individual was multiplied by the allele dose, and then, the scores of each SNP were accumulated to obtain the PRS of the individual (50). To get the effect values of the selected SNPs, we applied logistic regression to regress each SNP on BMD groups. The effect values were reported as the log odds ratios [ln(ORs)]. In the following formula, S represented the effect value, G represented the allele dose, subscript i represented the serial number of the SNP, and subscript j represented the individual serial number.


$$P\,R\,S_{j}=\sum_{i}S_{i}\times G_{i\,j}$$

### Dietary information collection and healthful plant-based diet index

A standardized questionnaire was conducted by trained interviewers to obtain data on dietary habits. The questionnaire includes information about the type of food, the frequency, and the average amount each time of consuming certain food in the past year. The type of food was divided into 10 categories (fresh vegetables, dessert, dairy, fresh aquatic food, fresh meat, staple, fresh fruit, pickled food, egg, and soybean product), and a subject’s annual intake of each food category was further assigned a value of 1–5 according to the quintile group it belongs to. Plant-based diets, mainly defined as “vegetarian” diets, have been associated with healthy (51). Studies have shown that under a plant-based diet, there is a risk of decreased BMD and increased fracture probability (37, 38). Therefore, food intake was estimated based on a healthful plant-based diet index (hPDI). It emphasized the intake of healthy plant-based foods related to improving health outcomes and divided the ingested foods into healthy plant-based foods, unhealthy plant-based foods, and animal-based foods. We ranked food intake from less to more, grouped healthy plant-based foods (fresh fruit, vegetables, and soybean product) into quintiles, and assigned them from 1 to 5 (51). The unhealthy plant-based foods (desserts, staples, and pickled food) and animal-based foods (dairy, fresh aquatic food, fresh meat, and egg) were also grouped according to the quintile, and the reverse values were assigned from 5 to 1 (51).

### Covariates

A detailed questionnaire and clinical examination provided information on covariates. Data such as demographic status (age and sex) and lifestyle (current cigarette smoking, current alcohol consumption, current tea consumption, and exercise frequency) were obtained *via* interviewer-administered questionnaires. According to a previous study, female sex and increasing age are predictors of low bone mass (52). Exercise activity was assessed by the frequency of exercise participation and categorized into “never,” “1–3 times per month,” “1–2 times per week,” “3–5 times per week,” and “6–7 times per month.” Participants’ waist and hip circumferences were collected through physical examination, and the WHR information was induced by dividing waist circumferences by hip circumferences. PRS and hPDI have been introduced above.

### Statistical analysis

For demographic, lifestyle, and metabolites, continuous variables were expressed as mean (standard deviation), and analysis of variance (ANOVA) was used to compare the differences among groups. Categorical variables were expressed in frequencies and compared using Pearson’s test. Multinomial logistic regression analysis was used to examine the relationship of each metabolite with BMD status. Interaction analysis was performed after standardizing the metabolites. We built a mediation effect model which used bootstrapping to test the mediation effect of metabolites on the correlation between lifestyles, PRS, and T-score (53–55). We used linear regression models to analyze the association between lifestyle and PRS and metabolites, as well as the association between metabolites and T-score. The analyses were performed using R (3.6.1, R core team), Stata/MP 16.0, and Cytoscape (v3.8.2). Two-tailed *p* < 0.05 was considered to indicate a statistically significant difference.

## Results

### Characteristics of study participants

Table 1 shows the characteristics of the study population across groups of normal BMD, osteopenia, and osteoporosis. The proportion of women in the osteoporosis group was significantly higher than that in the other two groups (87.80% vs. 62.80% vs. 33.60%, *p* < 0.001). The three groups differed in WHR, smoking, alcohol consumption, tea consumption, diabetes, and hPDI (*p* < 0.05).

**TABLE 1:** Characteristics of the study population by groups.

Variables	Normal BMD	Osteopenia	Osteoporosis	*P*-value
*N* (%)	312 (39.10)	353 (44.20)	132 (16.50)	–
Female, *n* (%)	105 (33.60)	222 (62.80)	116 (87.80)	**<0.001**
Age, years	59.56 (0.18)	59.97 (0.17)	60.09 (0.28)	0.141
WHR	0.92 (0.00)	0.90 (0.00)	0.90 (0.01)	**<0.001**
Smoker, *n* (%)	64 (20.50)	43 (12.10)	4 (3.00)	**<0.001**
Alcohol consumption, *n* (%)	126 (40.30)	91 (25.70)	10 (7.50)	**<0.001**
Tea consumption, *n* (%)	115 (36.80)	64 (18.10)	13 (9.80)	**<0.001**
Exercise frequency, *n* (%)	59 (18.91)	48 (13.60)	18 (13.64)	0.372
Diabetes, *n* (%)	39 (12.50)	32 (9.00)	9 (6.80)	**0.038**
hPDI	30.19 (0.24)	31.42 (0.24)	32.31 (0.38)	**<0.001**
PRS	0.66 (0.01)	0.68 (0.01)	0.65 (0.02)	0.23

Data represent number of the mean (standard error) or subjects (%). Bold values indicate significant P-values.

### Differential metabolites between groups

**FIGURE 2:**
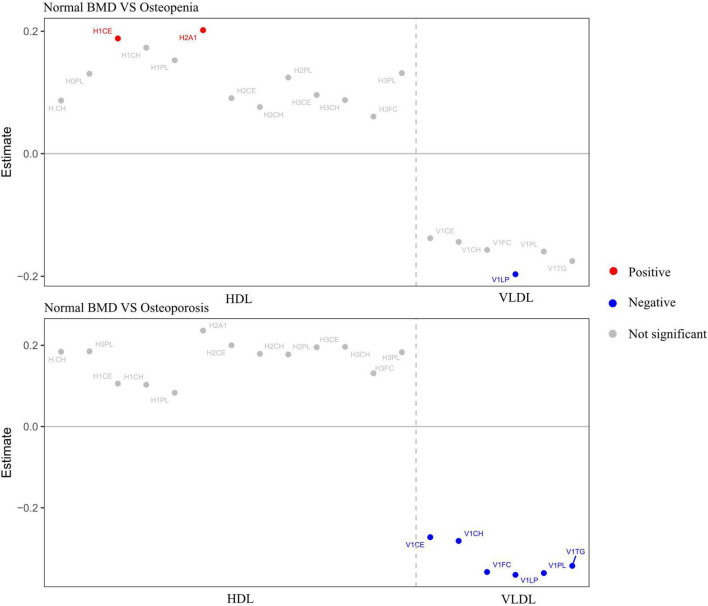
Metabolites with significant differences between groups. Adjusted for age, sex, WHR, and diabetes. Red dots represented positive correlation, blue dots represented negative correlation, and gray dots represented no significant correlation.

The metabolites included in this study were lipids, lipoproteins, amino acids, and other metabolites (see Supplementary Table 1 for details on the classification of metabolites). ANOVA was also used to explore the differential metabolites between the three BMD groups, and nineteen metabolites (subfractions of VLDL and subfractions of HDL) were significant between groups (FDR-adjusted *P* < 0.05). These 19 different metabolites were H-CH (HDL cholesterol), H0PL (phospholipids in HDL), H1CE (cholesterol esters in HDL-1), H1PL (phospholipids in HDL-1), H1CH (cholesterol in HDL-1), H2PL (phospholipids in HDL-2), H2A1 (Apo-A1 in HDL-2), H2CH (cholesterol in HDL-2), H2CE (cholesterol esters in HDL-2), H3CH (cholesterol in HDL-3), H3CE (cholesterol esters in HDL-3), H3PL (phospholipids in HDL-3), H3FC (free cholesterol in HDL-3), V1PL (phospholipids in VLDL-1), V1CE (cholesterol esters in VLDL-1), V1TG (triglycerides in VLDL-1), V1FC (free cholesterol in VLDL-1), V1CH (cholesterol in VLDL-1), and V1LP (total lipid in VLDL-1) and then performed multinomial logistic regression analysis on these 19 different metabolites; a total of eight metabolites were statistically significant. Figure 2 shows nineteen metabolites with significant differences between groups. The red dots represented positive correlation, the blue dots represented negative correlation, and the gray dots represented insignificant correlation.

### Associations between metabolites and different groups considering confounders

We further investigated the association between metabolites and different groups considering the intervention of confounders. Table 2 shows the significant results of multiple logistic regression analysis using metabolites as independent variables to compare the two groups with bone loss and the normal BMD group. After adjustment for age, sex, WHR, diabetes, smoking, tea consumption, alcohol consumption, hPDI, and PRS, H1CE (OR = 1.22, 95%CI: 1.01–1.48) and H2A1 (OR = 1.22, 95%CI: 1.01–1.48) were proved to be risk factors for bone loss, while V1LP (OR = 0.82, 95%CI: 0.68–0.99), V1LP (OR = 0.68, 95%CI: 0.52–0.89), V1TG (OR = 0.70, 95%CI: 0.53–0.91), V1PL (OR = 0.68, 95%CI: 0.52–0.90), V1FC (OR = 0.67, 95%CI: 0.49–0.90), V1CH (OR = 0.72, 95%CI: 0.55–0.96), and V1CE (OR = 0.73, 95%CI: 0.55–0.97) were protective factors for bone loss.

**TABLE 2:** Comparison of metabolites between groups.

Compared group	Metabolite	Model 1		Model 2		Model 3	
		Odds ratio (95%CI)	*p*-value	Odds ratio (95%CI)	*p*-value	Odds ratio (95%CI)	*p*-value
Osteopenia	H1CE	1.21 (1.01, 1.44)	**0.036**	1.22 (1.02, 1.45)	**0.030**	1.22 (1.01, 1.48)	**0.039**
Osteopenia	H2A1	1.22 (1.03, 1.46)	**0.025**	1.25 (1.04, 1.50)	**0.018**	1.22 (1.01, 1.48)	**0.040**
Osteopenia	V1LP	0.82 (0.68, 0.99)	**0.034**	0.84 (0.69, 1.01)	0.057	0.82 (0.68, 0.99)	**0.044**
Osteoporosis	V1LP	0.69 (0.54, 0.90)	**0.005**	0.70 (0.54, 0.91)	**0.007**	0.68 (0.52, 0.89)	**0.005**
Osteoporosis	V1TG	0.71 (0.55, 0.92)	**0.008**	0.72 (0.56, 0.93)	**0.012**	0.70 (0.53, 0.91)	**0.008**
Osteoporosis	V1PL	0.70 (0.54, 0.91)	**0.007**	0.70 (0.54, 0.92)	**0.009**	0.68 (0.52, 0.90)	**0.007**
Osteoporosis	V1FC	0.70 (0.53, 0.93)	**0.013**	0.70 (0.53, 0.93)	**0.015**	0.67 (0.49, 0.90)	**0.008**
Osteoporosis	V1CH	0.75 (0.58, 0.98)	**0.037**	0.76 (0.58, 0.99)	**0.046**	0.72 (0.55, 0.96)	**0.026**
Osteoporosis	V1CE	0.76 (0.58, 0.99)	**0.043**	0.77 (0.58, 1.01)	0.055	0.73 (0.55, 0.97)	**0.032**

Model 1 was adjusted for age, gender, WHR, and diabetes. Model 2 was adjusted for age, gender, WHR, diabetes, smoking, alcohol consumption, tea consumption, and hPDI. Model 3 was adjusted for age, gender, WHR, diabetes, smoking, alcohol consumption, tea consumption, hPDI, and PRS. Bold values indicate significant P-values.

### Analysis of the interaction effects

Previous reports suggested that the BMD-associated loci identified so far in GWAS still cannot account for all the heritability in osteoporosis (56). Consideration of gene–metabolite interactions might explain part of this “missing heritability” (56). To examine potential relationships between the nineteen differential metabolites and genes in BMD groups, we selected nine SNPs from two genes screened from three databases to calculate PRS (Supplementary Table 2). To investigate whether PRS modifies the association between BMD groups and metabolite concentrations, we tested for associations of nineteen differential metabolites and PRS. At the same time, we also explored the role of lifestyle in changing the association between the BMD group and metabolite. As shown in Table 3, there was a statistically significant interaction between PRS and V1CE, a subfraction of VLDL. However, the results of interaction analysis showed that PRS alone had no significant effect on the BMD group, but the interaction between PRS and V1CE was significant, indicating that there may be an interaction between PRS and V1CE to affect the BMD group. In addition, there was a statistically significant interaction between tea consumption and H3FC, which suggested that tea consumption and H3FC may interact with each other to affect the BMD group.

**TABLE 3:** Interaction effects in predicting osteoporosis risk.

Variables	Metabolite	Variable beta (95%CI)	Variable SE	Variable *p*-value	Interaction beta (95%CI)	Interaction SE	Interaction *p*-value
PRS	V1CE	0.18 (–0.51, 0.88)	0.35	0.61	0.76 (0.04, 1.48)	0.37	**0.037**
Tea consumption	H3FC	–0.41 (–0.81, –0.007)	0.21	**0.046**	–0.37 (–0.71, –0.03)	0.17	**0.032**

Adjusted by age, gender, diabetes, and WHR. Bold values indicate significant P-values.

### Analysis of the mediation effects of metabolites

**FIGURE 3:**
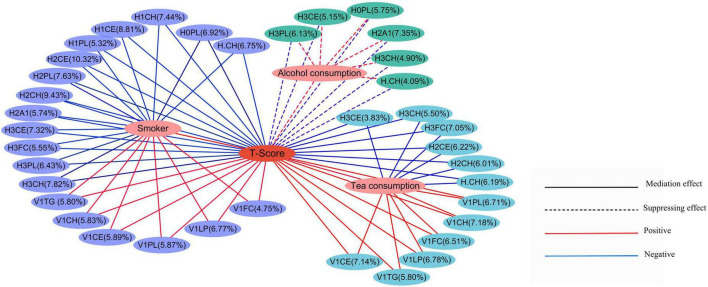
Mediation effects of lifestyle on T-score through the metabolites. The percentages showed the percentage of the intermediary variable explaining the correlation between the independent variable and the dependent variable. The solid lines represented the mediation effect, while the dashed lines represented the suppressing effects. The red lines represented a positive correlation, and the blue lines represented a negative correlation.

Furthermore, we carried out a mediation analysis to explore the effects of metabolites on the relationship between lifestyle and T-score. In the results of mediation analysis, the total effect was the effects of lifestyle factors (PRS, smoking, hPDI, alcohol consumption, and tea consumption) on T-score (without metabolites); the direct effect (ADE) was the effects of lifestyle factors on T-score after considering the mediation effect of metabolites; the mediation effect (ACME) was the total effect minus the direct effect (Supplementary Table 3). All direct effects, mediation effects, and total effects presented in Figure 3 were statistically significant. The results indicated that the effect of tea consumption on T-score was mediated by the metabolites: Increased tea consumption was positively associated with increased T-score which was mediated through levels of subfractions of HDL (H.CH, H2CH, H2CE, H3FC, H3CH, and H3CE) and VLDL (V1CH, V1PL, V1FC, V1CE, V1LP, and V1TG). In addition, the effect of smoking on T-score was mediated by the metabolites: The increase in smoking was positively correlated with the increase in T-score, and this increase was mediated by the levels of subfractions of HDL (H.CH, H0PL, H1CE, H1CH, H1PL, H2A1, H2CH, H2CE, H2PL, H3PL, H3FC, H3CH, and H3CE) and VLDL (V1CH, V1PL, V1FC, V1CE, V1LP, and V1TG). Besides, in the effect of alcohol consumption on the T-score, the direct effects and mediation effects had opposite signs, and the effect of alcohol consumption on the T-score was suppressed by the metabolites (55).

## Discussion

In this study, we analyze the association of metabolites and BMD considering the influence of genes and lifestyles in an elderly Chinese population. Mediation effects of the metabolites were found in the relationship between tea consumption, smoking, and T-score, and we found significant interactions between metabolites and tea consumption and PRSinn regulating BMD.

The association between metabolites and bone loss has been widely investigated (57–64). Previous studies have shown that high levels of HDL-C affect BMD through sex hormones including androgens and estrogen (57–60). According to related studies, combined oral estrogen/progestogen increased BMD in postmenopausal women but decreased HDL-C levels (61). Besides, VLDL has been proved positively correlated with bone mass. It has been pointed out that VLDL may cause atherosclerosis, and atherosclerosis was positively correlated with bone loss (62–64). In this study, we found that H1CE and H2A1 were positively correlated with bone loss. On the contrary, V1LP, V1TG, V1PL, V1FC, V1CH, and V1CE decreased the odds of osteoporosis or osteopenia compared to normal BMD.

To examine the potential effects of genes and lifestyles on the relations between the nineteen differential metabolites and BMD, we conducted an interaction analysis. There was a statistically significant interaction between PRS and V1CE, a subfraction of VLDL. However, the results of interaction analysis showed that PRS alone had no significant effect on the BMD group, but the interaction between PRS and metabolites was significant, indicating that PRS may affect the BMD group by affecting metabolites. The SNPs we selected from the three databases came from two genes: *LRP5* and *ESR1*. As an estrogen receptor, *ESR1* can affect the bone formation of osteoblast progenitor cells and mature osteoblasts (65, 66). A study has pointed out that estrogen can stimulate the production of VLDL (67). Combined with our research results, it is possible that the *ESR1* gene can affect VLDL by regulating estrogen and then affect BMD. *LRP5* is a member of the LDL receptor family, which also includes the VLDL receptor (68). As a lipoprotein receptor-related protein gene, the *LRP5* gene may affect BMD by regulating lipoprotein levels. During bone formation, *LRP5* is involved in upregulating transcription factors that are important for osteoblast differentiation (17). Of note, bone homeostasis is mainly controlled by the action of osteoblasts, osteocytes, and osteoclasts, which undergoes a continuous cycle of osteoblast-mediated bone formation and osteoclast-promoted bone resorption (56). The disruption of bone homeostasis plays a fundamental role in the pathogenesis of osteoporosis (69).

Drinking tea is one of the most common habits in the world, and tea is rich in antioxidants, which are good for human health. Current studies have shown that tea consumption could reduce the risk of osteoporosis (33). This may be related to the rich antioxidants in tea, which have been reported to have potentially beneficial effects on bone health (70, 71). We found that increased tea consumption was positively associated with increased T-score which was mediated through the levels of subfractions of HDL (H.CH, H2CH, H2CE, H3FC, H3CH, and H3CE) and VLDL (V1CH, V1PL, V1FC, V1CE, V1LP, and V1TG) in our results. Meanwhile, HDL has been negatively correlated with BMD, and VLDL has been positively correlated with BMD according to previous research, which is an effective support for our results (10, 72). A study has pointed out that long-term tea consumption is related to the reduction of HDL and may destroy the reverse cholesterol transport process mediated by HDL (73). However, there are considerable inconsistencies in the findings about the association between VLDL and tea consumption (74, 75). Compared with previous studies, our research found the mediation role of VLDL in the influence of tea consumption on the T-score. Of note, the result of interaction analysis showed that there was a significant interaction between H3FC and tea consumption. Our results showed that there was a significant correlation between tea drinking and the sub-components of HDL and VLDL, and tea drinking was positively correlated with the increase in T-score, indicating that tea drinking may be a protective factor for the decrease in bone mass.

The results of mediating effect analysis showed that there was a positive correlation between smoking and T-score, which was inconsistent with the results of previous studies (76, 77). Of the 797 subjects included in this study, only one female smokes, and the rest are men. In this study, the proportion of women in the bone mass reduction group and osteoporosis group is much higher than that in the normal bone mass group. Therefore, the correlation between smoking and T-score obtained in this study may be inconsistent with previous studies (76, 78).

Different intake of alcohol has different effects on bones. It is reported that a small amount of alcohol can increase BMD, but a large amount of alcohol can cause varying degrees of bone damage (14). Women drink one cup a day and men drink two cups a day, which is harmless to bone tissue, while higher alcohol consumption (2–4 cups a day) will damage bone tissue. The effect of drinking on bone may also be indirectly produced by reducing calorie intake and changing body composition (31). In our results, alcohol consumption was positively correlated with the T-score, which may be affected by the amount of alcohol. Our results showed that alcohol consumption was positively correlated with the level of HDL subfractions, which was consistent with the previous results (32). Our results showed that there was a significant difference in hPDI among the three BMD groups, and the hPDI in the osteoporosis group was significantly higher than that of the osteopenia group and normal BMD group. Previous studies have shown that under the plant-based diet, there is a risk of decreased BMD and an increased probability of fractures (25, 26). It has been proved that a plant-based diet reduces calcium and vitamin D intake and leads to an increase in N-telopeptide biomarkers, which is consistent with increased bone resorption (27). Plant-based diets are typically lower in saturated fatty acids, compared with omnivorous diets (79). The observed effects of plant-based diets on plasma lipoproteins may be the result of differences in saturated fatty acid intake (80, 81).

This study considered the effects of genes and lifestyles when analyzing the association between metabolites and BMD, and detailed interaction and mediation analyses were performed. However, several limitations in our study warrant mention. This study could not avoid selection bias, mainly as it selected Chinese volunteers from rural populations, which could have potentially influenced the results of the experiment. This is a cross-sectional research project, and it is not yet possible to predict the disease in osteoporosis through metabolites. However, the age of the subjects in our cohort was 55–65 years old, which was the stage from bone loss to osteoporosis. Through follow-up, we can better observe the trend of changes in BMD and research the progress of the disease. It is warranted to conduct longitudinal studies about lifestyle, metabolites, and BMD, which will provide more information about the therapeutic and preventive potentials of metabolites in osteoporosis. The TIS is an ongoing cohort, and data from the follow-ups will support this kind of research in future.

## Conclusion

Our research considered the effects of life factors and genes and explored the influence of metabolites on BMD. In summary, we found that polygenic risk scores and lifestyle can affect BMD by affecting metabolites. With the increased level of HDL subfractions, the risk of bone loss in the population will increase. The risk of bone loss decreases with the increased level of very-low-density lipoprotein subfractions. Extending our work to longitudinal data may eventually pave the way for the precise prevention of osteoporosis.

## Data availability statement

Data can be made available to interested researchers upon reasonable request.

## Ethics statement

This study received approval by the Ethics Committee of the School of Life Sciences, Fudan University, and Fudan University Taizhou Institute of Health Sciences (institutional review board approval number: 496 and B017, respectively). The patients/participants provided their written informed consent to participate in this study.

## Author contributions

KX and XL took part in the study design, analyzed, interpreted, and wrote the manuscript. YJ, DY, CZ, HY, ZY, and CS recruited the study participants and supported XL in the analysis of data. KX, XC, and YJ supervised the revision of the manuscript. All authors read and approved the final manuscript.
